# High-throughput DNA sequencing to survey bacterial histidine and tyrosine decarboxylases in raw milk cheeses

**DOI:** 10.1186/s12866-015-0596-0

**Published:** 2015-11-17

**Authors:** Daniel J. O’Sullivan, Vincenzo Fallico, Orla O’Sullivan, Paul L. H. McSweeney, Jeremiah J. Sheehan, Paul D. Cotter, Linda Giblin

**Affiliations:** Teagasc Food Research Centre, Moorepark, Fermoy, Cork, Ireland; School of Food and Nutritional Sciences, University College Cork, Cork, Ireland; Alimentary Pharmabiotic Centre, Cork, Ireland

**Keywords:** Biogenic amines, High-throughput sequencing, Histamine, Tyramine, Cheese microbiota, Ion PGM sequencing

## Abstract

**Background:**

The aim of this study was to employ high-throughput DNA sequencing to assess the incidence of bacteria with biogenic amine (BA; histamine and tyramine) producing potential from among 10 different cheeses varieties. To facilitate this, a diagnostic approach using degenerate PCR primer pairs that were previously designed to amplify segments of the histidine (*hdc*) and tyrosine (*tdc*) decarboxylase gene clusters were employed. In contrast to previous studies in which the decarboxylase genes of specific isolates were studied, in this instance amplifications were performed using total metagenomic DNA extracts.

**Results:**

Amplicons were initially cloned to facilitate Sanger sequencing of individual gene fragments to ensure that a variety of *hdc* and *tdc* genes were present. Once this was established, high throughput DNA sequencing of these amplicons was performed to provide a more in-depth analysis of the histamine- and tyramine-producing bacteria present in the cheeses. High-throughput sequencing resulted in generation of a total of 1,563,764 sequencing reads and revealed that *Lactobacillus curvatus*, *Enterococcus faecium* and *E. faecalis* were the dominant species with tyramine producing potential, while *Lb. buchneri* was found to be the dominant species harbouring histaminogenic potential. Commonly used cheese starter bacteria, including *Streptococcus thermophilus* and *Lb. delbreueckii,* were also identified as having biogenic amine producing potential in the cheese studied. Molecular analysis of bacterial communities was then further complemented with HPLC quantification of histamine and tyramine in the sampled cheeses.

**Conclusions:**

In this study, high-throughput DNA sequencing successfully identified populations capable of amine production in a variety of cheeses. This approach also gave an insight into the broader *hdc* and *tdc* complement within the various cheeses. This approach can be used to detect amine producing communities not only in food matrices but also in the production environment itself.

**Electronic supplementary material:**

The online version of this article (doi:10.1186/s12866-015-0596-0) contains supplementary material, which is available to authorized users.

## Background

High-throughput sequencing (HTS) has significantly enhanced our ability to profile complex microbial ecosystems such as those in the sea [1], soil [2], gut [3] and various foods including cheese [4–7]. While most of these studies rely on amplifying regions of the bacterial 16S rRNA or fungal ITS genes to study the microbial composition of these communities, it is also possible to use HTS to sequence select non-16S based genes [8]. With reference to this, HTS-based methods are currently being explored to improve food safety by targeting specific undesirable populations/genes [9, 10], and the potential exists to target genes involved in biogenic amine (BA) formation. BAs are low molecular weight organic bases with biological activity produced, primarily, by decarboxylation of precursor amino acids. BAs are classified according to their chemical structures and can be aromatic (tyramine), heterocyclic (histamine and tryptamine) or aliphatic (putrescine and cadaverine) [11–14]. In eukaryotes BAs are generally associated with a variety of biological processes including blood pressure regulation, neurotransmission, cellular growth and allergic responses. In prokaryotes, however, BA formation is generally linked with cell survival, particularly in low pH conditions where it serves as a stress response mechanism. Up-regulation of decarboxylase gene expression has previously been shown to occur in the presence of the precursor amino acid and in low pH environments, such as those encountered in fermented foods. The amino acid/amine transporter system also acts to generate energy in the form of proton motive force, thus providing a further competitive advantage under such stress conditions [15, 16]. Microbial BA formation is encountered in a variety of fermented foods and beverages including cheese, fish, beer, wine, meat products and fermented vegetables [17]. The most commonly occurring BAs detected in foods include histamine, tyramine, putrescine and cadaverine [18]. The accumulation of histamine and/or tyramine at high levels may produce toxicological effects including hypertension, headaches, palpitations and vomiting in certain individuals, particularly those with reduced mono/di-amine oxidase activity, due to either genetic or pharmacological reasons. The European Food Safety Authority regard histamine and tyramine as the most important BAs from a toxicological viewpoint [19]. Additionally, the presence of di-amines, such as putrescine and cadaverine, can further promote toxicological effects as they act as potentiators of histamine and tyramine toxicity by competing for detoxifying enzymes [20–24]. As the detrimental effects associated with consumption of BAs varies depending on the amine in question and the susceptibility of the individual, it is particularly difficult to set defined limits for BAs in food products [25]. Consequently, regulatory limits describing BA concentrations have yet to be established for the cheese industry. Notably, ripened cheeses are second only to fish as the most commonly implicated source of dietary BAs [19, 26, 27], which has led to the coining of the term the “cheese reaction” [28].

BAs can be formed by a variety of cheese associated lactic acid bacteria (LAB) including *Lactobacillus*, *Lactococcus*, *Streptococcus*, *Leuconostoc* and *Enterococcus* [15, 17, 18, 23]. Several factors are associated with the accumulation of BAs in cheese including low pH, milk processing parameters (raw/pasteurised), the presence of amine forming species (starter or non-starter/contaminating bacteria), availability of precursor amino acids, ripening temperature/time and salt content, among other factors [29]. While the majority of cheese is produced from pasteurised milk, raw milk cheeses are also popular due to their unique flavour characterisitics [27]. High levels of secondary proteolysis as a result of starter and non-starter bacterial action, together with higher microbial load and, in many cases, long ripening times make raw milk cheeses particularly susceptible to BA formation [13, 14, 27, 28, 30, 31]. The presence of BAs can also be used as an indicator of overall product hygiene in the form of biogenic amine indices [19].

Methods employed to detect BAs in dairy products have been extensively reviewed [15, 20, 29, 32, 33]. Essentially, detection is either direct, i.e., detection of the respective amines or indirect, i.e., based on identifying amine forming bacteria. Amine detection methods rely primarily on chromatographic techniques such as thin layer and high performance liquid chromatography (HPLC) [29]. While initial approaches for identifying responsible bacteria were based on differential chromogenic agars and enzymatic methods, more recently, molecular based methods such as DNA hybridisation, polymerase chain reaction (PCR) and quantitative (q)PCR have been used [20, 32, 34]. PCR based approaches are of particular use for establishing the aminogenic potential of various isolates from food products. In this instance, strains associated with raw materials, production equipment and, in the case of cheese, starter bacteria can be pre-emptively screened for decarboxylase biomarkers leading to a potential reduction of amines in the final product. A review published by Landete et al [20] describes several sets of PCR primers for detecting producers of the major food-associated amines [20].

In this study a range of raw milk, speciality cheeses were screened for the presence of histidine decarboxylase (*hdc*) and tyrosine decarboxylase (*tdc*) genes associated with the production of histamine and tyramine, respectively. Previously optimised PCR primer pairs amplifying regions of the Gram-positive *hdc* and *tdc* gene clusters were employed and the resultant amplicons were cloned and subjected to Sanger sequencing in order to establish that that there was sufficient heterogeneity among the decarboxylases present to merit a more detailed HTS analysis. HTS revealed the dominant and sub-dominant species with tyramine and histamine producing potential, in these raw milk cheeses. More importantly, the value of employing HTS to survey decarboxylase genes within a microbial population is established.

## Methods

### Sample collection

Ten speciality cheeses were purchased from a local market. Raw milk cheeses with long ripening times (3 – 24 months) were selected and divided into 2 groups (hard and semi-hard). Cheeses originated from several European countries including two Irish artisanal cheeses (A and B), Reblochon, Manchego, Morbier, Tête de Moine, Pecorino Sardo, Ossau-Iraty, Comté and Gorgonzola. Cheeses were vacuum packed and stored at 4 °C for 3 days prior to DNA extraction. Table 1 provides a description of the cheeses selected for this study. These particular cheeses were selected due to their potential to accumulate BAs and are not reflective of all cheese within the respective categories.

**Table 1:** Description of cheeses

Cheese	Milk Type and Source	Age	Type	Region	Rind	Total BA by HPLC (mg/kg)	*Hdc* gene presence by PCR	*Tdc* gene presence by PCR
Irish Artisanal Cheese A	Raw, Cow	12 – 18 months	Hard	Ireland	Waxed	290.3	N	Y
Reblochon	Raw, Cow	4 - 12 weeks	Semi-hard	France	Washed, smear ripened	104.1	Y	Y
Irish Artisanal Cheese B	Raw, Cow	12 - 18 months	Hard	Ireland	Cloth bound natural	456.6	Y	Y
Manchego	Raw, Sheep	6 -12 months	Semi-hard	Spain	Waxed	21.9	N	N
Morbier	Raw, Cow	2 – 3 months	Semi-hard	France	Natural	736.5	Y	N
Tête de Moine	Raw, Cow	3 – 6 months	Hard	Switzerland	Washed	131.9	Y	Y
Pecorino Sardo	Raw, Sheep	6 – 10 months	Hard	Italy	Natural	134.2	Y	Y
Ossau-Iraty	Raw, Sheep	3 – 6 months	Semi-hard	France	Natural	393.8	Y	Y
Comté	Raw, Cow	6 – 12 months	Hard	France	Natural	13.8	N	N
Gorgonzola	Raw, Cow	3 – 4 months	Semi-hard	Italy	Natural	34.2	N	N

Description of cheeses including age, origin and rind type. HPLC results as well as presence of the respective decarboxylases detected by PCR are also included

### Determination of BA content of cheese

BAs were acid extracted, derivatised and quantified, in duplicate, using the method described by Özoğul [35] with modifications for a cheese matrix. Five grams of cheese was weighed into a sterile bag containing 20 ml 0.013 N H_2_SO_4_. The suspension was homogenised in a stomacher (Iul Instruments, Barcelona, Spain) for 10 min. The liquid phase was transferred to a sterile 50 ml tube while the remaining cheese homogenate was subjected to a second acid extraction with 20 ml 0.013 N H_2_SO_4_. The liquid phases were pooled and centrifuged at 5000 g, 4 °C for 15 min. After centrifugation, the solution was brought to a final volume of 50 ml with 0.013 N H_2_SO_4_. A 10 ml aliquot was filtered using 0.2 μm cellulose acetate filters (Chromacol, Welwyn Garden, Herts, UK).

Extracted BAs were then derivatised by mixing 1 ml of each respective extract with 1 ml 2 N NaOH and 1 ml 2 % benzoyl chloride (Sigma-Aldrich, Wicklow, Ireland) in glass test tubes. The mixture was vortexed and allowed to stand for 15 min prior to the addition of 2 ml saturated NaCl. Two ml of diethyl ether was then added. A plastic pipette was used to transfer the top layer of the extract to a second glass test tube with a further 2 ml diethyl ether added and the resultant top layers pooled. Diethyl ether was evaporated off using a stream of nitrogen at 45 °C for 20 min. The BA residue was dissolved by adding 1 ml acetonitrile.

BAs were separated using a Luna C18 RF 5 μm, 100 Å column 250 x 4.6 mm (Phenomenex Queens Avenue, Macclesfield, UK) and were eluted at an initial flow rate of 1.6 ml/min for 30 min with Acetonitrile (A) and H_2_O (B), using the following gradients:

- 0-1 min 1.6 ml/min 40 % A + 60 % B
- 1-10 min 1.8 ml/min 50 % A + 50 % B
- 10-20 min 2.0 ml/min 60 % A + 40 % B
- 20-25 min 2.0 ml/min 70 % A + 30 % B
- 25-26 min 1.6 ml/min 40 % A + 60 % B
- 26-30 min 1.6 ml/min 40 % A + 60 % B

BAs were quantified using 5 data points on calibration curves against standard solutions of histamine (100-2000 μg/ml), tyramine (5-100 μg/ml), putrescine and cadaverine (Sigma-Aldrich, Dublin, Ireland) (Additional file 1: Table S1). Data was presented as mg of individual BA per kg of cheese.

### Determination of cheese pH, salt and moisture contents

Grated samples of each cheese were analysed for salt content [36], moisture [37] and pH [38] using previously described methods.

### DNA extraction from selected cheeses

Five grams of each cheese was homogenised in 45 ml of a 2 % tri-sodium citrate buffer (VWR, Dublin, Ireland). Cheese homogenate was then subjected to enzymatic lysis using lysozyme (1 mg/ml), mutanolysin (50 U/ml) (Sigma Aldrich, Dublin, Ireland) and proteinase k (800 μg/ml) and incubated at 55 °C for 30 min as per Quigley et al [39]. DNA was extracted using the PowerFood Microbial DNA Isolation Kit (MoBio Laboratories Inc, Carlsbad, CA USA). After extraction, DNA was concentrated via ethanol precipitation. DNA was re-suspended in 20 μl TE buffer (Sigma-Aldrich, Dublin, Ireland). Quality and purity of extracted DNA was assessed using the NanoDrop 1000 Spectrophotometer (Thermo-Fisher Scientific, Wilmington, VA, USA), as per manufacturers guidelines.

### PCR detection of *hdc* and *tdc* gene fragments using selected primer sets

PCR based detection of decarboxylase genes was achieved using primers specific for regions of the Gram-positive and Gram-negative *hdc* operon, respectively, as well as for the *tdc* operon. Primers for the *hdc* operon of Gram-positive bacteria comprised of a forward (HDC3 5’- GATGGTATTGTTTCKTATGA-3’) and a reverse primer (HDC4 5’ CAAACACCAGCATCTTC-3’) targeting a 435 bp fragment of the *hdcA* gene [18]. Primers targeting the Gram-negative *hdc* operon comprised of a forward (HIS2-F 5’-AAYTSNTTYGAYTTYGARAARGARGT-3’) and a reverse primer (HIS2-R 5’-TANGGNSANCCDATCATYTTRTGNCC-3’), and generated a 531 bp product [40]. The *tdc* primers, comprised of a forward (TD5 ‘5- CAAATGGAAGAAGAAGTAGG-3’) and a reverse primer (TD2 ‘5- ACATAGTCAACCATRTTGAA-3’), amplified an 1100 bp fragment of the *tdc* gene as described by Coton et al [24]. PCR reactions were carried out in triplicate and contained 25 μl BioMix Red Master Mix (Bioline, London, UK), 1 μl of each primer (200 nmol l^-1^), 5 μl DNA template (standardised to 100 ng DNA/reaction) and nuclease free water to a final volume of 50 μl. PCR amplification was carried out using a G-Storm Thermal Cycler (Gene Technologies, Oxfordshire, UK). Amplification consisted of an initial denaturation at 95 °C for 10 min followed by 40 cycles of; denaturation at 95 °C for 45 s, annealing at 48 °C for 1 min and extension at 72 °C for 90 s. This was followed by a final elongation step at 72 °C for 7 min. PCR amplicons were pooled and cleaned using the AMPure XP magnetic bead-based purification system (Beckman Coulter, Takeley, UK).

### Cloning of PCR amplicons

Cleaned PCR amplicons were subjected to TOPO cloning reactions using the TOPO TA cloning kit (Invitrogen, CA, USA). TOP10 *E. coli* (Invitrogen) were transformed with the resultant plasmids and plated on LB agar (Merck) containing 50 μg/ml kanamycin (Sigma Aldrich, Dublin, Ireland). Transformants were selected from each cloning reaction and cultured overnight in LB broth and 50 μg/ml kanamycin. Plasmids were then extracted from overnight cultures using the QIAprep Spin Mini Prep kit (Qiagen, Crawley, Sussex, UK) according to the manufacturer’s guidelines. Extracted plasmids were quantified and assessed for quality using the NanoDrop 1000 Spectrophotometer (Thermo-Fisher Scientific, Wilmington, VA, USA) prior to Sanger sequencing (Source BioSciences, Dublin, Ireland). The *hdc* amplicons were sequenced using the M13 forward primer while *tdc* amplicons were sequenced using both the M13 forward and reverse primers supplied with the TOPO TA cloning kit.

### High throughput sequencing

Prior to HTS, *tdc* amplicon libraries were prepared using the Ion Xpress Plus Fragment Library Kit (Life Technologies, Dublin, Ireland). The *hdc* libraries, for which fragmentation was not required, were prepared using the Ion Plus Fragment Library Kit (Life Technologies, Dublin, Ireland). Libraries were then barcoded, prior to sequencing, using the Ion Xpress Barcode Adaptors (Life Technologies, Dublin, Ireland). Amplicons libraries were assessed for size distribution and concentration using a Bioanalyser 2100 (Agilent Technologies, Santa Clara, CA USA). Following library quantification and equimolar pooling, the Ion OneTouch 2 system was used to prepare template positive Ion Sphere Particles (ISP) containing the clonally amplified DNA libraries using the Ion PGM Template OT2 400 kit which allows for < 400 bp reads. Enrichment of the template positive ISP’s was performed using the Ion OneTouch ES. An enrichment percentage of 18 % was obtained. Sequencing was performed on the Ion Torrent PGM (Life Technologies, Dublin, Ireland) using an Ion 318v2 chip and the Ion PGM Sequencing 400 kit (Life Technologies, Dublin, Ireland) at the Teagasc Next Generation Sequencing suite as per the manufacturer’s guidelines.

### Bioinformatic analysis

Following Sanger sequencing, *hdc* reads were analysed using the NCBI nucleotide database (BlastN; http://blast.ncbi.nlm.nih.gov/). Sanger sequencing of the *tdc* amplicons did not provide forward and reverse reads of the complete 1100 bp, therefore, only the overlap (approximately 800 bp), aligned using the MegAlign programme was analysed using the BlastN database.

Raw Ion PGM reads were quality filtered with the fastq_filter script in USEARCH. For both *tdc* and *hdc* amplicons, a length cut-off of 170 bp was used. Reads were then clustered into operational taxonomical units (OTUs) and chimeras removed with the 64-bit version of USEARCH [41]. Subsequently OTUs were aligned with MUSCLE [42] and a phylogenetic tree generated within Qiime [43]. Alpha diversity analysis was also implemented within Qiime. For taxonomic assignment OTUs were blasted against the NCBI-Nr database and parsed through MEGAN [44].

## Results

This study used previously published PCR primers, designed based on alignments of conserved regions of decarboxylase gene clusters from known BA producing isolates [20]. In order to be sure that the variety of decarboxylase genes within the selected cheeses was sufficiently heterogeneous to merit culture-independent HTS analysis, an initial Sanger sequencing-based investigation of cloned PCR amplicons was undertaken. This was then followed by HTS to profile the dominant and subdominant histamine and tyramine producing populations present in the respective cheeses.

### Sanger sequencing reveals the identity of bacteria with histaminogenic potential

The selected *hdc* primers targeted a 435 bp fragment of the Gram-positive *hdcA* gene. Six of the 10 cheeses sampled generated PCR amplicons corresponding to the *hdc* operon (Reblochon, Irish artisanal cheese B, Morbier, Tête de Moine, Pecorino Sardo, Ossau-Iraty). No amplicons were generated, across all cheese varieties, when using the selected Gram-negative *hdc* primers [20]. The Gram-positive *hdc* amplicons were cloned via the TOPO TA cloning method and a subset of 46 clones were subjected to Sanger sequencing. Table 2 contains a summary of BLAST output for each cheese sample while Additional file 1: Table S2 contains a complete BLAST analysis of each respective cheese including scores generated, query cover and accession numbers. BLAST output indicated that 35 of the 46 clones sequenced (76.1 %) contained a *hdc* fragment corresponding to the *Lactobacillus buchneri hdc* operon. Other *hdc* sequences identified corresponded to the *hdc* operon that is conserved across *Lactobacillus sakei/Tetragenococcus halophilus/T. muriaticus/Oenococcus oeni/Lactobacillus hilgardii hdc* operon (hereafter referred to as the *Lb. sakei* group of *hdc* operon; 23.4 %). In the Reblochon and Tête de Moine cheeses, all of the sequenced *hdc* clones (8 and 8, respectively) corresponded to the *Lb. buchneri hdc* operon. In the Ossau-Iraty cheese all of the *hdc* positive clones were identified as corresponding to the *hdc* operon of the *Lb. sakei* group. The *hdc* genes from *Lb. buchneri* and the *Lb. sakei* group were identified from among the Irish artisanal cheese B, Morbier and Pecorino Sardo cheeses while clones corresponding to the *Lb. sakei* group *hdc* operon were identified from among the Ossau-Iraty cheese.

**Table 2:** Summary of histidine decarboxylase BLAST analysis

Cheese	# of Clones	BLAST output	E-value	% Identity
Reblochon	8	*Lb. buchneri* histidine decarboxylase operon (*hdcA* gene, *hdcB* gene, *hdcC* gene and *hisS* gene)	0	99 %
Irish Artisanal Cheese B	5	*Lb. buchneri* histidine decarboxylase operon (*hdcA* gene, *hdcB* gene, *hdcC* gene and *hisS* gene)	0	99 %
	1	*Lb.sakei* hdc gene, partial cds/*T. halophilus hdc* operon/*T. muriaticus* p*hdc/O. oeni hdc* operon*/Lb. hilgardii hdc* operon	0	99 %
Morbier	7	*Lb. buchneri* histidine decarboxylase operon (hdcA gene, hdcB gene, hdcC gene and hisS gene)	0	99 %
	1	*Lb.sakei* hdc gene, partial cds/*T. halophilus hdc* operon/*T. muriaticus* p*hdc/O. oeni hdc* operon*/Lb. hilgardii hdc* operon	0	96 %
Tête De Moine	8	*Lb. buchneri* histidine decarboxylase operon (*hdcA* gene, *hdcB* gene, *hdcC* gene and *hisS* gene)	0	99 %
Pecorino Sardo	7	*Lb. buchneri* histidine decarboxylase operon (*hdcA* gene, *hdcB* gene, *hdcC* gene and *hisS* gene)	0	99 %
	1	*Lb.sakei* hdc gene, partial cds/*T. halophilus hdc* operon/*T. muriaticus* p*hdc/O. oeni hdc* operon*/Lb. hilgardii hdc* operon	0	99 %
Ossau-Iraty	8	*Lb.sakei* hdc gene, partial cds/*T. halophilus hdc* operon/*T. muriaticus* p*hdc/O. oeni hdc* operon*/Lb. hilgardii hdc* operon	0	99 %

Summary of homologues of histidine decarboxylase (*hdc*) gene fragments detected in *hdc* positive cheeses using Sanger sequencing of cloned amplicons

### Sanger sequencing reveals the identity of bacteria with tyraminogenic potential

PCR amplification, using primers designed based on alignments of tyrosine decarboxylases from known producers [20], detected the presence of an 1100 bp fragment of the *tdc* gene in 6 of the 10 cheeses tested (Irish artisanal cheese A, Reblochon, Irish artisanal cheese B, Tête de Moine, Pecorino Sardo, Ossau-Iraty). Table 3 depicts a summary of the BLAST output for each positive cheese samples while Additional file 1: Table S3 contains a complete BLAST analysis of samples including top hits, scores generated, query cover and accession numbers. Resultant amplicons were cloned and subjected to Sanger sequencing. In this instance, a subset of 44 clones was sequenced across the six positive cheese types. BLAST analysis revealed the presence of *tdc* fragments corresponding to several species, including *Enterococcus faecalis* which accounted for 19 of the 44 clones sequenced (43.1 %). The *tdc* fragments from *Lactobacillus curvatus/Streptococcus thermophilus* (which share high identity with one another; 36 %)*, E. faecium* (18 %) and *Lactobacillus plantarum/brevis* (which, again, are not easily distinguished; 2.3 %) were also identified across the 6 cheese types. With respect to the Pecorino Sardo cheese, all clones contained *tdc* genes corresponding to that of and *E. faecium*. In contrast, *tdc* genes corresponding to those of enterococci, streptococci and lactobacilli were detected across all other cheese varieties.

**Table 3:** Summary of tyrosine decarboxylase BLAST analysis

Cheese	# of Clones	Gene target	BLAST output	E-value	% Identity
Irish Artisanal Cheese A	5	*tdc*	*E. faecalis tdc* operon complete cds	0	99 %
	1	*tdc*	*E. faecalis tdc* operon complete cds	1.0E-141	99 %
	2	*tdc*	*Lb. curvatus tdc* complete cds/*S. thermophilus tdcA* gene	0	99 %
Reblochon	5	*tdc*	*Lb. curvatus tdc* /*S. thermophilus tdcA* gene complete cds	0	99 %
	1	*tdc*	*E. faecalis tdc* gene, complete cds	0	100 %
Irish Artisanal Cheese B	8	*tdc*	*Lb. curvatus tdc*, complete cds/*S. thermophilus tdcA* gene	0	99 %
Tête de Moine	7	*tdc*	*E. faecalis tdc* gene, complete cds	0	98 %
	1	*tdc*	*Lb. plantarum/Lb. brevis tdc* gene cds	0	99 %
Pecorino Sardo	6	*tdc*	*E. faecium tyrS gene, tyrdc* gene complete cds	0	99 %
	1	*tdc*	*E. faecium tyrS gene, tyrdc* gene complete cds	2E-70	79 %
	1	*tdc*	*E. faecium tyrS gene, tyrdc* gene complete cds	0	89 %
Ossau-Iraty	2	*tdc*	*E. faecalis*, *tdc* gene complete cds	0	98 %
	2	*tdc*	*E. faecalis* complete genome	0	97 %
	1	*tdc*	*E. faecalis* complete genome	0	99 %
	1	*tdc*	*Lb. curvatus tdc* gene complete cds/*S. thermophilus tdcA* gene	0	99 %

Summary of homologues of tyrosine decarboxylase (*tdc*) gene fragments detected in *tdc* positive cheeses using Sanger sequencing of cloned amplicons

### α-diversity of artisanal cheese microbiota with BA-producing potential as revealed by next generation DNA sequencing

Sanger sequencing established that several cheese samples contained multiple microbial sources of decarboxylase genes. As a result it was apparent that the use of a culture-independent HTS-based approach to provide an in-depth insight into the diversity of the populations present was justified. The previously generated PCR amplicons were used for HTS sequencing (n = 6 for gram-positive *hdc* primers and n = 6 for *tdc* primers). Amplicons were subjected to HTS using the Ion PGM platform, generating 938,971 *hdc* reads and 624,793 *tdc* reads, after quality filtering (refer to Additional file 1: Table S4 for the complete list of assigned reads/cheese). Mean read length across both *tdc* and *hdc* samples was 245 bp. Operational Taxonomic Unit (OTU) diversity (α-diversity) was calculated for both *hdc* and *tdc* samples and is displayed in Table 4. For *hdc* α-diversity, Chao1 values, indicative of taxonomic richness, ranged from 41.75 – 90 while the Shannon index, used to measure the overall sample diversity of Gram-positive bacteria with histamine-producing potential, ranged from 2.57 – 3.23. Irish artisanal cheese B displayed the greatest sample diversity while Tête de Moine exhibited the lowest diversity. The *hdc* α-diversity was observed to be lower than that of the *tdc* samples. For *tdc* samples, Chao1 values ranged from 224.25 – 279.62 while the Shannon index ranged from 5.48 – 6.4. Ossau-Iraty displayed the greatest sample diversity while Irish artisanal cheese B displayed the lowest sample diversity. The phylogenetic diversity value and number of observed OTU matrices also indicated that α-diversity was considerably greater in *tdc* samples than *hdc* samples.

**Table 4:** α-diversity post Ion PGM sequencing in *hdc* and *tdc* positive cheese samples

*hdc* α-diversity					
Cheese	Chao1 value	Simpson value	Shannon index value	Phylogenetic diversity value	No. of observed OTU’s
Reblochon	55	0.80	2.85	21.96	52
Irish Artisanal Cheese B	90	0.82	3.23	27.71	75
Morbier	57.5	0.76	2.73	20.38	57
Tête de Moine	41.75	0.67	2.39	18.25	38
Pecorino Sardo	69.5	0.75	2.73	21.66	67
Ossau-Iraty	52	0.78	2.57	23.48	50
*tdc* α-diversity					
Cheese	Chao1 value	Simpson value	Shannon index value	Phylogenetic diversity value	No. of observed OTU’s
Irish Artisanal Cheese A	249.06	0.98	6.40	145.48	246
Reblochon	247.96	0.97	5.48	143.93	225
Irish Artisanal Cheese B	224.25	0.97	5.51	126.47	188
Tête de Moine	273.50	0.97	5.78	171.53	270
Pecorino Sardo	270.18	0.97	5.81	152.71	259
Ossau-Iraty	279.62	0.98	5.96	150.62	256

α-diversity of artisanal cheeses post Ion PGM sequencing. Table 4a details diversity of *hdc* positive samples while Table 4b presents *tdc* positive samples

### High-throughput Ion PGM sequencing reveals the presence of amine forming communities in different cheese varieties

**Fig. 1:**
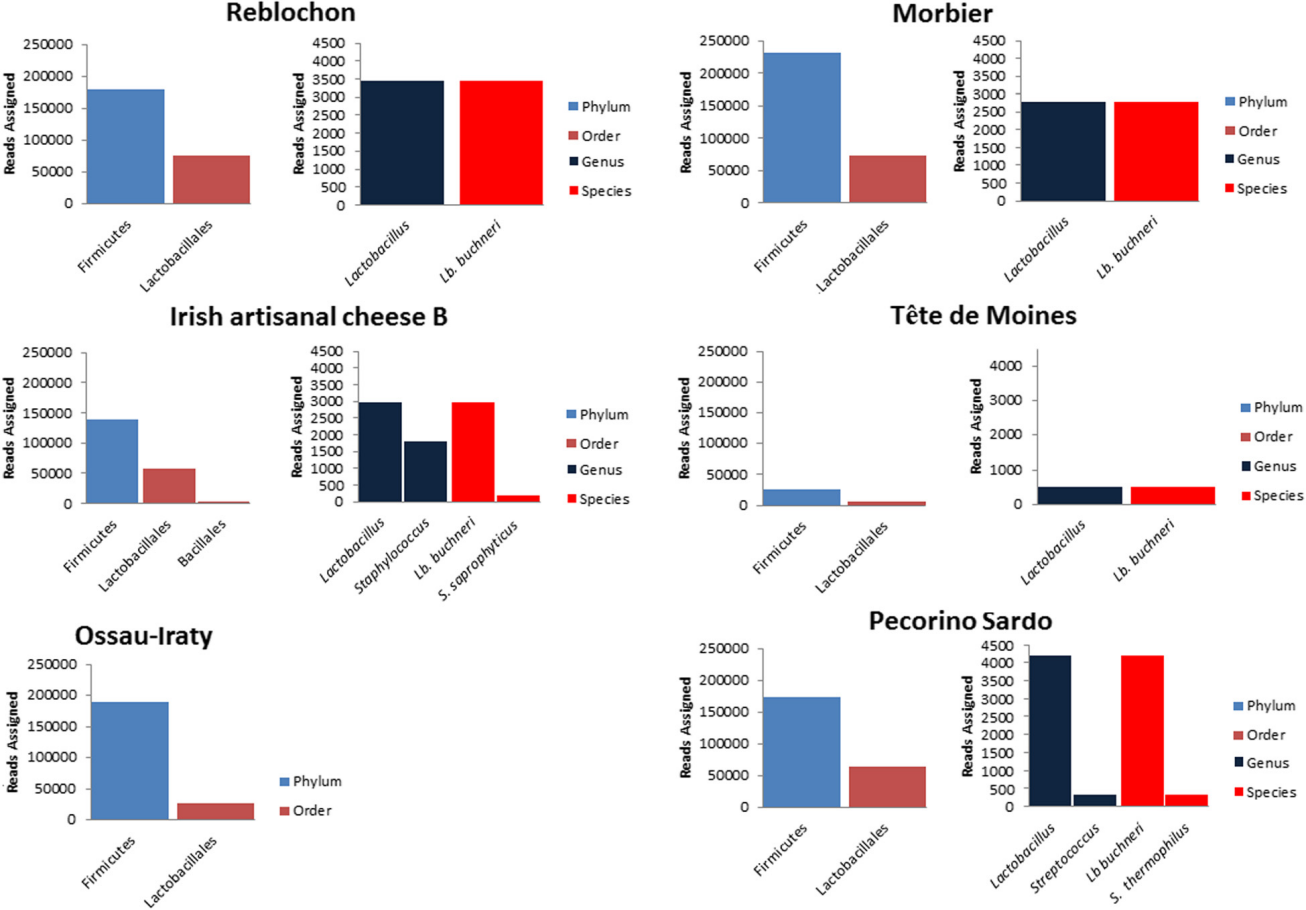
Phylogenetic assignement, using MEGAN, of *hdc* reads across 10 speciality cheeses at Phylum, Order, Genus and Species level. Note that no genus or species level assignement was possible for the Ossau-Iraty cheese

**Fig. 2:**
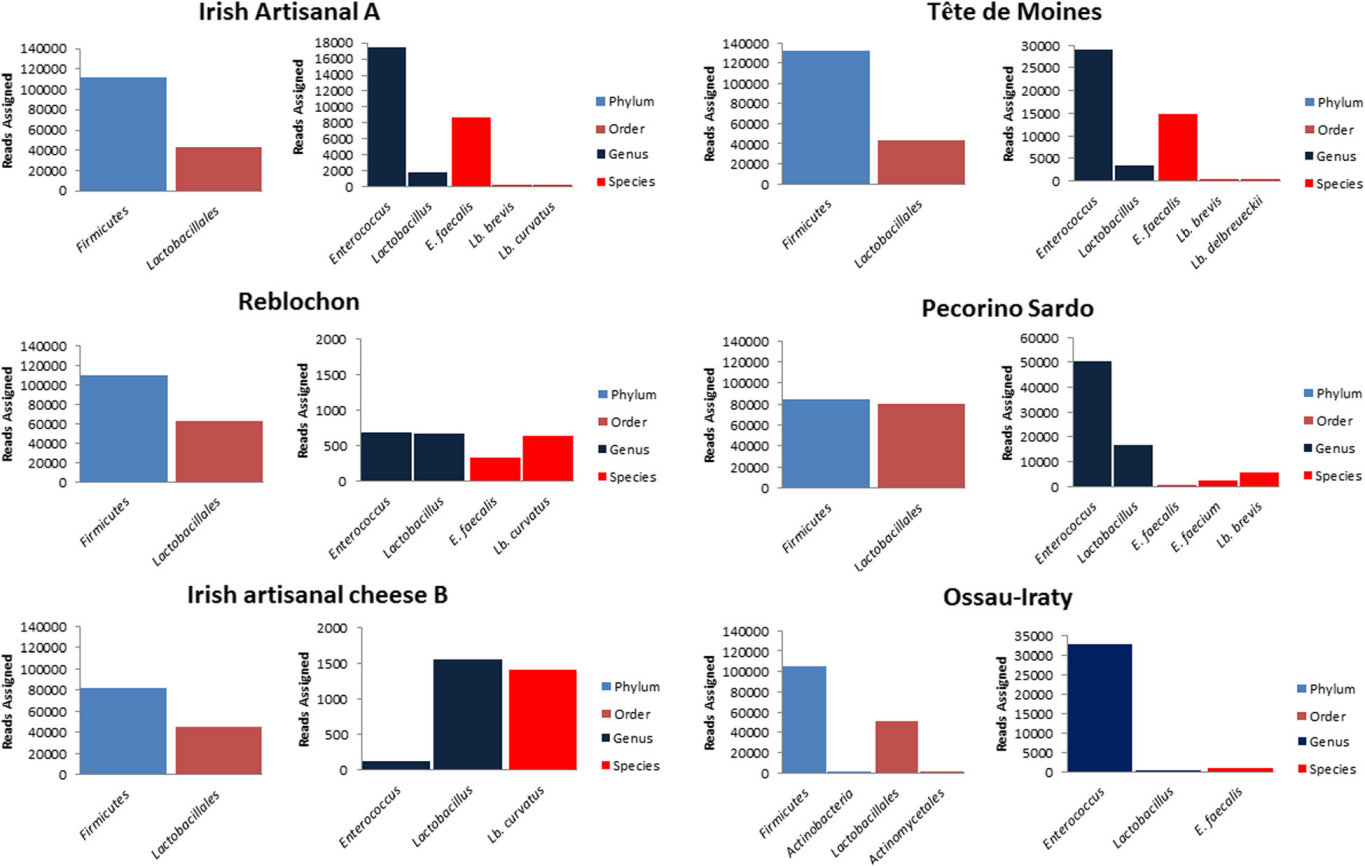
Phylogenetic assignement, using MEGAN, of *tdc* reads across 10 speciality cheeses at Phylum, Order, Genus and Species level

Phylogenetic assignment of high-throughput sequence data revealed *tdc* sequences corresponding to representatives of both the Firmicutes (99.84 – 100 % of all *tdc* sequences) and Actinobacteria (0.16 % of *tdc* sequences) phyla. All the *hdc* sequences belonged to the Firmicutes phylum (Additional file 1: Table S5a/b). The small proportion of *tdc* reads assigned to the phylum Actinobacteria corresponded to the cheese Ossau-Iraty. While reads were successfully allocated at phylum level, there was an expected, progressive reduction in the numbers of assigned reads at order, genus and species levels respectively. Reads successfully allocated, at phylum, order, genus and species levels, are displayed in Figs. 1 and 2. At the order level, *Lactobacillales* accounted for 33.14 – 95.11 % of reads assigned in the *tdc* samples. The Actinobacteria-assigned *tdc* reads in Ossau-Iraty corresponded to *Actinomycetales* at the order level and to *Micrococcinaeae* at family level but could not be assigned at the genus level. With respect to the *hdc* samples, *Lactobacillales* accounted for 13.7 – 42.3 % of the reads assigned at the order level.

At the genus and species levels, the numbers of reads that could be unambiguously assigned was low in all cases (depicted in Additional file 1: Table S4) and this was particularly evident when analysing the *hdc* samples. With respect to *hdc* samples, *Lactobacillus* accounted for 62.5 % to 100 % of all reads assigned at the genus level. Populations corresponding to *Staphylococcus* (37.5 % of reads assigned at genus level) were present in Irish artisanal cheese B, while *Streptococcus* (6.93 % of reads assigned at genus level) was identified in the Pecorino Sardo cheese. At the species level, a small cohort of the *Staphylococcus* population was identified as *S. saprophyticus* (5.97 % of reads successfully assigned at species level) while *Streptococcus* populations were successfully classified as *S. thermophilus* (6.94 % of reads successfully assigned at species level). *Lb. buchneri* accounted for the majority of reads assigned (93.06 – 100 %) at species level and was detected across all cheeses except for Ossau-Iraty (Fig. 1). With respect to the Ossau-Iraty cheese, no genus or species level assignment was possible.

For the *tdc* samples, reads were assigned primarily to the genus *Enterococcus* and ranged from 7.67 – 99.65 % of reads assigned at genus level. *Lactobacillus* populations were also present and accounted for 0.35 – 92.33 % of reads assigned at genus level. At the species level, *E. faecalis* accounted for the majority (2.29 - 100 %) of reads successfully assigned at species level. Other subdominant populations identified included *E. faecium, Lb. curvatus, Lb. brevis* and *Lb. delbrueckii* (Fig. 2). Percentage populations of reads assigned exclusively at genus and species levels are shown in Additional file 1: Table S6.

### Cheese characterisation

BAs were detected, at various concentrations, in all cheeses sampled and were found to range from 13.8 – 736.5 mg/kg (Table 5). The average histamine content of the positive samples was 34.48 mg/kg while the average tyramine concentration was 108.69 mg/kg. In all cases more than one BA was present in the cheeses sampled. Although not as toxicologically important as histamine and tyramine, putrescine and cadaverine levels were also measured to give a total BA concentration in each cheese. As expected, tyramine, generally regarded as the most common BA present in cheese [16, 19], was present in 9 cheese samples at concentrations ranging from 4.5 to 323.4 mg/kg. Histamine was present in 8 cheeses (8.4 – 85.1 mg/kg). Cadaverine was detected in all cheese samples at concentrations ranging from 1.2 – 267.4 mg/kg, while putrescine was detected in 7 cheeses (3.9 – 212.7 mg/kg). The presence or concentration of BAs in the respective cheeses did not appear to be influenced by milk type, source or age. The Morbier cheese contained the highest concentration of total BAs (736.5 mg/kg) while the Comté cheese contained only 13.8 mg/kg total BAs. Histamine was not detected by HPLC in the Manchego and Comté cheeses. Similarly, tyramine was not detected in the Gorgonzola cheese by HPLC.

**Table 5:** BA concentrations detected in cheese samples

Cheese	Histamine (mg/kg)	Tyramine (mg/kg)	Putrescine (mg/kg)	Cadaverine (mg/kg)	Total BA (mg/kg)
Irish Artisanal Cheese A	22.9	140.4	122.0	5.0	290.3
Reblochon	8.4	45.1	28.2	22.3	104.1
Irish Artisanal Cheese B	34.4	190.6	157.2	74.4	456.6
Manchego	n.d.	17.9	n.d.	4.0	21.9
Morbier	85.1	171.3	212.7	267.4	736.5
Tête de Moine	51.6	44.6	n.d.	35.7	131.9
Pecorino Sardo	23.4	40.4	66.9	3.5	134.2
Ossau-Iraty	20.8	323.4	40.1	9.4	393.8
Comté	n.d.	4.5	n.d.	9.3	13.8
Gorgonzola	29.2	n.d.	3.9	1.2	34.2

Average concentrations of biogenic amines (mg/kg of cheese) detected as determined by HPLC

Compositional analyses of the cheeses are presented in Table 6. Salt concentrations ranged from 0.65 – 1.99 %, while cheese pH values extended from 5.3 to 7.1. Cheese salt in moisture levels ranged from 2.1 to 6.48.

**Table 6:** Cheese compositional analysis

Cheese	Salt (%)	pH	Salt in moisture levels
Irish Artisanal Cheese A	1.59	5.3	6.26
Reblochon	1.08	6.4	2.10
Irish Artisanal Cheese B	1.99	5.4	6.48
Manchego	1.44	5.7	5.24
Morbier	1.36	6.9	4.32
Tête de Moine	1.49	7.1	4.46
Pecorino Sardo	1.72	5.6	6.44
Ossau-Iraty	1.42	6.4	4.73
Comté	0.65	6.1	2.34
Gorgonzola	1.96	7.1	4.32

Compositional analysis of cheeses (Salt %, pH and Salt in Moisture)

## Discussion

In this study, a novel, targeted sequencing-based approach was used to screen a range of different cheese varieties for the presence of microbial populations capable of producing the major toxic BAs histamine and tyramine. Initially, Sanger sequencing identified common BA producers (*Lb. buchneri*, *E. faecium* and *E. faecalis*) [23, 45] but more importantly provided proof of heterogeneity justifying the use of NGS. The longer read lengths associated with the Sanger approach (up to approximately 800 bp in the case of the *tdc* amplicon) also allowed, in certain instances, successful identification at genus and species levels. However, the highly conserved nature of decarboxylase genes often reduced the capacity for distinguishing between certain species. This was particularly evident with respect the *Lb. sakei*/*T. halophilus*/*T. muriaticus*/*O.oeni/Lb. hilgardii hdc* operons and the *Lb. curvatus/S. thermophilus* and *Lb. plantarum/Lb. brevis tdc* operons identified. In the aforementioned cases, when conducting a BLAST analysis, the query cover and % identity are identical while the maximum scores differ slightly. This is as a result of single nucleotide changes in the analysed sequences (described in Additional file 1: Tables S2 and S3). In the case of the *Lb. curvatus/S. thermophilus tdc* operons identified, it likely that both of these cheese associated species are present within the samples tested. With respect to the difficulty differentiating *Lb. sakei/T. halophilus/T. muriaticus/O. oeni/Lb. hilgardii hdc* operons, it is difficult to predict the exact species present.

A further 1,563,764 sequence reads were generated by high-throughput DNA sequencing of amplicons (post quality filtering). HTS allowed for greater population coverage but, in many cases, the short read length led to reduced resolution. Decarboxylases from common BA producers such as *E. faecalis, Lb. buchneri*, *Lb. brevis*, and *Lb. curvatus* were again identified. Subdominant populations, for example *Staphylococcus saprophyticus* and *Lactobacillus delbrueckii*, which were not observed *via* Sanger sequencing, were also present at less than 1 % of total reads. The shorter read lengths (mean read length of 245 bp) associated with using high-throughput sequencing, meant that, in some cases, the assignment of reads at genus and species levels was challenging (Figs. 1 and 2). This is particularly relevant with respect to the highly conserved *hdc* operon. The absence of decarboxylase gene specific databases, as compared to the well annotated 16S rRNA databases, also affected the identification by BLAST analysis. Thus the combination of reduced read length and the lack of specific databases reduced the identification capacity of the HTS-based approach. This issue is particularly noticeable when analysing the microbial composition of the raw sheep milk cheese Ossau-Iraty. With reference to Ossau-Iraty, Sanger sequencing allowed for successful identification of genes assigned to *E. faecalis, Lb. curvatus/S. thermophilus* (both *tdc*), and *Lb. sakei/T. halophilus/T. muriaticus/O. oeni/Lb. hilgardii* (*hdc*), however the high-throughput approach did not permit assignment of the *hdc* samples at the genus or species level. In the case of *tdc* analysis, the identification of *E. faecalis*-associated *tdc* was possible. Furthermore, while deep sequencing allowed the identification of *tdc* genes corresponding to *Actinomycetales* (0.16 %) (Fig. 2), which were assigned to the *Micrococcinaeae*, the shorter read length prevented assignment of these decarboxylases at genus or species levels.

HPLC results established the presence of various BAs across all cheeses sampled. However, the presence of histamine and/or tyramine did not always correlate with the presence of the corresponding decarboxylase gene fragment. This was most evident in the case of the Morbier cheese, which exhibited the highest total BA concentration in this study. Despite a tyramine concentration of 171.3 mg/kg, no *tdc* amplicons were generated by PCR. This discrepancy may be attributable to the fact that the primers selected for this study were designed to target Gram-positive LAB and were based on alignments with common (type-strains) species including *Lb. sakei*, *Lb. buchneri*, *Lactobacillus* 30a, *O. oeni*, *C. perfringens* and *T. muraticus* (*hdc*) and *Lb. brevis*, *C. divergens*, *C. piscicola*, *E. faecalis* and *E. faecium* (*tdc*) [18, 24]. Therefore, the primers may not bind to all histamine and tyramine decarboxylase determinants present within the cheeses. Indeed, certain yeast species including strains of *Y. lipolytica* (*tdc*), *D. hansenii* and *G. candidum* (*hdc*) are recognised BA producers associated with artisanal cheeses, and may have contributed to the amine content, but would not be detected using the primers employed [13].

In this study, the identification of decarboxylase genes, using HTS, from bacteria commonly used as cheese starter cultures, including *Lb. delbrueckii* and *S. thermophilus* was of particular interest [46]. In agreement with previous reports [23, 47], *S. thermophilus* was identified as having histidine decarboxylation capacity in the Pecorino Sardo cheese. The origin of these bacteria, i.e., whether they were added as cheese starters or gained access to the cheese *via* raw milk or during processing or ripening is not known. This highlights the importance of screening starter and adjunct bacteria for aminogenic potential, using molecular methods that can rapidly detect the presence of decarboxylase genes. *S. saprophyticus,* not commonly associated with BA formation in cheese, was identified in this study and has previously been associated with BA formation in fermented meat products [48, 49].

Of the cheeses selected for this study, both Pecorino-Sardo and Manchego have a well-established association with BAs. In particular, Pecorino Sardo, identified in this study as containing several *hdc* and *tdc* positive bacteria (*Lb. buchneri, E. faecium, E. faecalis*), has previously been shown to contain conditions (microbiota, ripening time, physio-chemical factors) complementary to BA production [30, 50]. Manchego has also previously been shown to contain tyrosine decarboxylating microorganisms; however, in this study the Manchego cheese sampled had a low level of total BA concentrations (21.9 mg/kg) and no *tdc* or *hdc* positive amplicons were generated [51]. Comté and Gorgonzola have also previously been shown to contain various BAs [52] but in our study BA levels were low and no *hdc* or *tdc* amplicons were generated. Interestingly, blue cheeses such as Gorgonzola are often regarded as having optimal conditions for BA production, due to milk processing and proteolytic activity (presence of molds), for BA formation, however, in this study the Gorgonzola sample exhibited among the lowest total BA concentrations [33, 53].

## Conclusions

Ultimately, this study shows, for the first time, that sequencing based technologies (Ion PGM platform) can successfully profile the diversity of histaminogenic and tyraminogenic bacteria present in ripening cheese. A similar approach could also be applied to reduce risk factors associated with BA accumulation. This can be achieved by screening starter cultures, milk and manufacturing/storage facilities with a view to reducing/controlling not only populations associated with BA formation, but potential sources of these populations [13, 54–56]. In this way, a pre-emptive approach using existing (refrigeration, preservatives, additives) and/or emerging (microbial modelling, high hydrostatic pressure, irradiation) control measures can be implemented [54, 57–60]. This method cannot, however, determine the transcriptional activity of the respective genes. In addition, while NGS reads indicate, proportionally, the levels of bacterial populations within the cheese matrix, it does not accurately quantify the numbers of bacteria present. While further optimisation is required, sequencing based approaches have the potential to eventually replace labour intensive culture-based methods which often require primary culturing followed by molecular methods to identify responsible genera.

## Additional file

Additional file 1:
**Table S1**. Standards preparation for HPLC analysis of individual biogenic amines. **Table S2.** Complete BLAST analysis of clones subjected to Sanger sequencing. **Table S3.** Table S2: Complete BLAST analysis of *tdc* clones subjected to Sanger sequencing. **Table S4.** Total reads assigned for each cheese. **Table S5a/b.** Microbial composition of bacteria at phylum, order, genus and species levels. **Figure S6.** Microbial composition at Genus and Species levels. (DOCX 62 kb)

## Abbreviations

BA: Biogenic amine
DNA: Deoxyribonucleic acid
*Hdc*: Histidine decarboxylase gene
*Tdc*: Tyrosine decarboxylase gene
PCR: Polymerase chain reaction
PGM: Personal genome machine
HTS: High throughput sequencing
ITS: Internal transcribed spacer
LAB: Lactic acid bacteria
HPLC: High performance liquid chromatography
N: Normal
TE: Tris ethylenediaminetetraacetic acid
BLAST: Basic local alignment search tool
OTU: Operational taxonomic unit
Bp: Base pair
