# Supplementary material for: High-throughput DNA sequencing to survey bacterial histidine and tyrosine decarboxylases in raw milk cheeses
Source: BMC Microbiol. 2015 Nov 17;15:266. doi: 10.1186/s12866-015-0596-0 (PMC4650399; doi:10.1186/s12866-015-0596-0)
Supplement: Additional file 1: — Table S1. Standards preparation for HPLC analysis of individual biogenic amines. Table S2. Complete BLAST analysis of clones subjected to Sanger sequencing. Table S3. Table S2: Complete BLAST analysis of tdc clones subjected to Sanger sequencing. Table S4. Total reads assigned for each cheese. Table S5a/b. Microbial composition of bacteria at phylum, order, genus and species levels. Figure S6. Microbial composition at Genus and Species levels. (DOCX 62 kb) [file 12866_2015_596_MOESM1_ESM.docx]

Supplementary Tables and Figures

Supplementary Table 1: Standards preparation for HPLC analysis of individual biogenic amines

| **Mix No.** | **Histamine (μg/ml)** | **Putrescine (μg/ml)** | **Cadaverine (μg/ml)** | **Tyramine (μg/ml)** |
| --- | --- | --- | --- | --- |
| 1 | 100 | 5 | 10 | 50 |
| 2 | 200 | 10 | 20 | 100 |
| 3 | 500 | 25 | 50 | 250 |
| 4 | 1000 | 50 | 100 | 500 |
| 5 | 2000 | 100 | 200 | 1000 |

Table S1: Composition of reference standard mixes used for HPLC quantification of biogenic amines in cheese

Supplementary Table 2: Complete BLAST analysis of clones subjected to Sanger sequencing

| **Cheese** | **Clone** | **Top BLAST Hits** | **Max score** | **Query cover** | **E value** | **Identity (%)** | **Accession** |
| --- | --- | --- | --- | --- | --- | --- | --- |
| Reblochon | 1 | *Lb. buchneri* *hdc* gene, partial cds | 798 | 100% | 0 | 99% | DQ132890.1 |
|  |  | *Lb. buchneri* *hdc* operon | 798 | 100% | 0 | 99% | AJ749838.1 |
| Reblochon | 2 | *Lb. buchneri* *hdc* gene, partial cds | 798 | 100% | 0 | 99% | DQ132890.1 |
|  |  | *Lb. buchneri* *hdc* operon | 798 | 100% | 0 | 99% | AJ749838.1 |
| Reblochon | 3 | *Lb. buchneri* *hdc* gene, partial cds | 793 | 100% | 0 | 99% | DQ132890.1 |
|  |  | *Lb. buchneri* *hdc* operon | 793 | 100% | 0 | 99% | DQ132890.1 |
| Reblochon | 4 | *Lb. buchneri* *hdc* gene, partial cds | 793 | 100% | 0 | 99% | DQ132890.1 |
|  |  | *Lb. buchneri* *hdc* operon | 793 | 100% | 0 | 99% | DQ132890.1 |
| Reblochon | 5 | *Lb. buchneri* *hdc* gene, partial cds | 798 | 100% | 0 | 99% | DQ132890.1 |
|  |  | *Lb. buchneri* *hdc* operon | 798 | 100% | 0 | 99% | AJ749838.1 |
| Reblochon | 6 | *Lb. buchneri* *hdc* gene, partial cds | 793 | 100% | 0 | 99% | DQ132890.1 |
|  |  | *Lb. buchneri* *hdc* operon | 793 | 100% | 0 | 99% | DQ132890.1 |
| Reblochon | 7 | *Lb. buchneri* *hdc* gene, partial cds | 793 | 100% | 0 | 99% | DQ132890.1 |
|  |  | *Lb. buchneri* *hdc* operon | 793 | 100% | 0 | 99% | DQ132890.1 |
| Reblochon | 8 | *Lb. buchneri* *hdc* gene, partial cds | 798 | 100% | 0 | 99% | DQ132890.1 |
|  |  | *Lb. buchneri* *hdc* operon | 798 | 100% | 0 | 99% | AJ749838.1 |
| Irish Artisanal Cheese B | 1 | *Lb. buchneri* *hdc* gene, partial cds | 793 | 100% | 0 | 99% | DQ132890.1 |
|  |  | *Lb. buchneri* *hdc* operon | 793 | 100% | 0 | 99% | AJ749838.1 |
| Irish Artisanal Cheese B | 2 | *Lb. buchneri* *hdc* gene, partial cds | 791 | 100% | 0 | 99% | DQ132890.1 |
|  |  | *Lb. buchneri* *hdc* operon | 791 | 100% | 0 | 99% | AJ749838.1 |
| Irish Artisanal Cheese B | 3 | *Lb. buchneri* *hdc* gene, partial cds | 787 | 100% | 0 | 99% | DQ132890.1 |
|  |  | *Lb. buchneri* *hdc* operon | 787 | 100% | 0 | 99% | AJ749838.1 |
| Irish Artisanal Cheese B | 4 | *Lb. buchneri* *hdc* gene, partial cds | 793 | 100% | 0 | 99% | DQ132890.1 |
|  |  | *Lb. buchneri* *hdc* operon | 793 | 100% | 0 | 99% | AJ749838.1 |
| Irish Artisanal Cheese B | 5 | *Lb. sakei hdc operon* | 782 | 100% | 0 | 99% | DQ132888.1 |
|  |  | *Lb. sakei* strain *hdc* gene, partial cds | 782 | 100% | 0 | 99% | AY800122.1 |
|  |  | *T. halophilus hdc* gene *c*omplete and partial cds | 776 | 100% | 0 | 99% | AB670117.1 |
|  |  | *O. oeni Hdc operon* | 776 | 100% | 0 | 99% | DQ132887.1 |
|  |  | *Lb. hilgardii hdc* operon | 776 | 100% | 0 | 99% | NG_036021.1 |
| Irish Artisanal Cheese B | 6 | *Lb. buchneri* *hdc* gene, partial cds | 798 | 100% | 0 | 99% | DQ132890.1 |
|  |  | *Lb. buchneri* *hdc* operon | 798 | 100% | 0 | 99% | AJ749838.1 |
| Morbier | 1 | *Lb. buchneri* *hdc* gene, partial cds | 713 | 100% | 0 | 100% | DQ132890.1 |
|  |  | *Lb. buchneri* *hdc* operon | 713 | 100% | 0 | 100% | AJ749838.1 |
| Morbier | 2 | *Lb. buchneri* *hdc* gene, partial cds | 787 | 100% | 0 | 99% | DQ132890.1 |
|  |  | *Lb. buchneri* *hdc* operon | 787 | 100% | 0 | 99% | AJ749838.1 |
| Morbier | 3 | *Lb. buchneri* *hdc* gene, partial cds | 787 | 100% | 0 | 99% | DQ132890.1 |
|  |  | *Lb. buchneri* *hdc* operon | 787 | 100% | 0 | 99% | AJ749838.1 |
| Morbier | 4 | *Lb. buchneri* *hdc* gene, partial cds | 798 | 100% | 0 | 99% | DQ132890.1 |
|  |  | *Lb. buchneri* *hdc* operon | 798 | 100% | 0 | 99% | AJ749838.1 |
| Morbier | 5 | *Lb. buchneri* *hdc* gene, partial cds | 782 | 100% | 0 | 99% | DQ132890.1 |
|  |  | *Lb. buchneri* *hdc* operon | 782 | 100% | 0 | 99% | AJ749838.1 |
| Morbier | 6 | *Lb. sakei hdc operon* | 699 | 100% | 0 | 96% | DQ132888.1 |
|  |  | *Lb. sakei* strain *hdc* gene, partial cds | 699 | 100% | 0 | 96% | AY800122.1 |
|  |  | *T. halophilus hdc* gene *c*omplete and partial cds | 693 | 100% | 0 | 95% | AB670117.1 |
|  |  | *T. muriaticus* plasmid pHDC-I-1 DNA, complete sequence | 693 | 100% | 0 | 95% | AB710473.1 |
|  |  | *O. oeni Hdc operon* | 693 | 100% | 0 | 95% | DQ132887.1 |
|  |  | *Lb. hilgardii hdc* operon | 693 | 100% | 0 | 95% | NG_036021.1 |
| Morbier | 7 | *Lb. buchneri* *hdc* gene, partial cds | 787 | 100% | 0 | 99% | DQ132890.1 |
|  |  | *Lb. buchneri* *hdc* operon | 787 | 100% | 0 | 99% | AJ749838.1 |
| Morbier | 8 | *Lb. buchneri* *hdc* gene, partial cds | 793 | 100% | 0 | 99% | DQ132890.1 |
|  |  | *Lb. buchneri* *hdc* operon | 793 | 100% | 0 | 99% | DQ132890.1 |
| Tête De Moine | 1 | *Lb. buchneri* *hdc* gene, partial cds | 798 | 100% | 0 | 99% | DQ132890.1 |
|  |  | *Lb. buchneri* *hdc* operon | 798 | 100% | 0 | 99% | AJ749838.1 |
| Tête De Moine | 2 | *Lb. buchneri* *hdc* gene, partial cds | 798 | 100% | 0 | 99% | DQ132890.1 |
|  |  | *Lb. buchneri* *hdc* operon | 798 | 100% | 0 | 99% | AJ749838.1 |
| Tête De Moine | 3 | *Lb. buchneri* *hdc* gene, partial cds | 793 | 100% | 0 | 99% | DQ132890.1 |
|  |  | *Lb. buchneri* *hdc* operon | 793 | 100% | 0 | 99% | DQ132890.1 |
| Tête De Moine | 4 | *Lb. buchneri* *hdc* gene, partial cds | 787 | 100% | 0 | 99% | DQ132890.1 |
|  |  | *Lb. buchneri* *hdc* operon | 787 | 100% | 0 | 99% | AJ749838.1 |
| Tête De Moine | 5 | *Lb. buchneri* *hdc* gene, partial cds | 787 | 100% | 0 | 99% | DQ132890.1 |
|  |  | *Lb. buchneri* *hdc* operon | 787 | 100% | 0 | 99% | AJ749838.1 |
| Tête De Moine | 6 | *Lb. buchneri* *hdc* gene, partial cds | 798 | 100% | 0 | 99% | DQ132890.1 |
|  |  | *Lb. buchneri* *hdc* operon | 798 | 100% | 0 | 99% | AJ749838.1 |
| Tête De Moine | 7 | *Lb. buchneri* *hdc* gene, partial cds | 798 | 100% | 0 | 99% | DQ132890.1 |
|  |  | *Lb. buchneri* *hdc* operon | 798 | 100% | 0 | 99% | AJ749838.1 |
| Tête De Moine | 8 | *Lb. buchneri* *hdc* gene, partial cds | 793 | 100% | 0 | 99% | DQ132890.1 |
|  |  | *Lb. buchneri* *hdc* operon | 793 | 100% | 0 | 99% | DQ132890.1 |
| Pecorino Sardo | 1 | *Lb. buchneri* *hdc* gene, partial cds | 798 | 100% | 0 | 99% | DQ132890.1 |
|  |  | *Lb. buchneri* *hdc* operon | 798 | 100% | 0 | 99% | AJ749838.1 |
| Pecorino Sardo | 2 | *Lb. buchneri* *hdc* gene, partial cds | 798 | 100% | 0 | 99% | DQ132890.1 |
|  |  | *Lb. buchneri* *hdc* operon | 798 | 100% | 0 | 99% | AJ749838.1 |
| Pecorino Sardo | 3 | *Lb. buchneri* *hdc* gene, partial cds | 793 | 100% | 0 | 99% | DQ132890.1 |
|  |  | *Lb. buchneri* *hdc* operon | 793 | 100% | 0 | 99% | DQ132890.1 |
| Pecorino Sardo | 4 | *Lb. buchneri* *hdc* gene, partial cds | 787 | 100% | 0 | 99% | DQ132890.1 |
|  |  | *Lb. buchneri* *hdc* operon | 787 | 100% | 0 | 99% | AJ749838.1 |
| Pecorino Sardo | 5 | *Lb. sakei hdc* operon | 787 | 100% | 0 | 99% | DQ132888.1 |
|  |  | *Lb. sakei* strain *hdc* gene, partial cds | 787 | 100% | 0 | 99% | AY800122.1 |
|  |  | *T. halophilus hdc* gene *c*omplete and partial cds | 782 | 100% | 0 | 99% | AB670117.1 |
|  |  | *T. muriaticus* plasmid pHDC-I-1 DNA, complete sequence | 782 | 100% | 0 | 99% | AB710473.1 |
|  |  | *O. oeni Hdc operon* | 782 | 100% | 0 | 99% | DQ132887.1 |
|  |  | *Lb. hilgardii hdc* operon | 782 | 100% | 0 | 99% | NG_036021.1 |
| Pecorino Sardo | 6 | *Lb. buchneri* *hdc* gene, partial cds | 798 | 100% | 0 | 99% | DQ132890.1 |
|  |  | *Lb. buchneri* *hdc* operon | 798 | 100% | 0 | 99% | AJ749838.1 |
| Pecorino Sardo | 7 | *Lb. buchneri* *hdc* gene, partial cds | 798 | 100% | 0 | 99% | DQ132890.1 |
|  |  | *Lb. buchneri* *hdc* operon | 798 | 100% | 0 | 99% | AJ749838.1 |
| Pecorino Sardo | 8 | *Lb. buchneri* *hdc* gene, partial cds | 798 | 100% | 0 | 99% | DQ132890.1 |
|  |  | *Lb. buchneri* *hdc* operon | 798 | 100% | 0 | 99% | AJ749838.1 |
| Ossau-Iraty | 1 | *Lb. sakei hdc* operon | 798 | 100% | 0 | 99% | DQ132888.1 |
|  |  | *Lb. sakei* strain *hdc* gene, partial cds | 798 | 100% | 0 | 99% | AY800122.1 |
|  |  | *T. halophilus hdc* gene *c*omplete and partial cds | 793 | 100% | 0 | 99% | AB670117.1 |
|  |  | *T. muriaticus* plasmid pHDC-I-1 DNA, complete sequence | 793 | 100% | 0 | 99% | AB710473.1 |
|  |  | *O. oeni Hdc operon* | 793 | 100% | 0 | 99% | DQ132887.1 |
|  |  | *Lb. hilgardii hdc* operon | 793 | 100% | 0 | 99% | NG_036021.1 |
| Ossau-Iraty | 2 | *Lb. sakei hdc* operon | 798 | 100% | 0 | 99% | DQ132888.1 |
|  |  | *Lb. sakei* strain *hdc* gene, partial cds | 798 | 100% | 0 | 99% | AY800122.1 |
|  |  | *T. halophilus hdc* gene *c*omplete and partial cds | 793 | 100% | 0 | 99% | AB670117.1 |
|  |  | *T. muriaticus* plasmid pHDC-I-1 DNA, complete sequence | 793 | 100% | 0 | 99% | AB710473.1 |
|  |  | *O. oeni Hdc operon* | 793 | 100% | 0 | 99% | DQ132887.1 |
|  |  | *Lb. hilgardii hdc* operon | 793 | 100% | 0 | 99% | NG_036021.1 |
| Ossau-Iraty | 3 | *Lb. sakei hdc* operon | 793 | 100% | 0 | 99% | DQ132888.1 |
|  |  | *Lb. sakei* strain *hdc* gene, partial cds | 793 | 100% | 0 | 99% | AY800122.1 |
|  |  | *T. halophilus hdc* gene *c*omplete and partial cds | 787 | 100% | 0 | 99% | AB670117.1 |
|  |  | *T. muriaticus* plasmid pHDC-I-1 DNA, complete sequence | 787 | 100% | 0 | 99% | AB710473.1 |
|  |  | *O. oeni Hdc operon* | 787 | 100% | 0 | 99% | DQ132887.1 |
|  |  | *Lb. hilgardii hdc* operon | 787 | 100% | 0 | 99% | NG_036021.1 |
| Ossau-Iraty | 4 | *Lb. sakei hdc* operon | 798 | 100% | 0 | 99% | DQ132888.1 |
|  |  | *Lb. sakei* strain *hdc* gene, partial cds | 798 | 100% | 0 | 99% | AY800122.1 |
|  |  | *T. halophilus hdc* gene *c*omplete and partial cds | 793 | 100% | 0 | 99% | AB670117.1 |
|  |  | *T. muriaticus* plasmid pHDC-I-1 DNA, complete sequence | 793 | 100% | 0 | 99% | AB710473.1 |
|  |  | *O. oeni Hdc operon* | 793 | 100% | 0 | 99% | DQ132887.1 |
|  |  | *Lb. hilgardii hdc* operon | 793 | 100% | 0 | 99% | NG_036021.1 |
| Ossau-Iraty | 5 | *Lb. sakei hdc* operon | 795 | 100% | 0 | 99% | DQ132888.1 |
|  |  | *Lb. sakei* strain *hdc* gene, partial cds | 795 | 100% | 0 | 99% | AY800122.1 |
|  |  | *T. halophilus hdc* gene *c*omplete and partial cds | 789 | 100% | 0 | 99% | AB670117.1 |
|  |  | *T. muriaticus* plasmid pHDC-I-1 DNA, complete sequence | 789 | 100% | 0 | 99% | AB710473.1 |
|  |  | *O. oeni Hdc operon* | 789 | 100% | 0 | 99% | DQ132887.1 |
|  |  | *Lb. hilgardii hdc* operon | 789 | 100% | 0 | 99% | NG_036021.1 |
| Ossau-Iraty | 6 | *Lb. sakei hdc* operon | 798 | 100% | 0 | 99% | DQ132888.1 |
|  |  | *Lb. sakei* strain *hdc* gene, partial cds | 798 | 100% | 0 | 99% | AY800122.1 |
|  |  | *T. halophilus hdc* gene *c*omplete and partial cds | 793 | 100% | 0 | 99% | AB670117.1 |
|  |  | *T. muriaticus* plasmid pHDC-I-1 DNA, complete sequence | 793 | 100% | 0 | 99% | AB710473.1 |
|  |  | *O. oeni Hdc operon* | 793 | 100% | 0 | 99% | DQ132887.1 |
|  |  | *Lb. hilgardii hdc* operon | 793 | 100% | 0 | 99% | NG_036021.1 |
| Ossau-Iraty | 7 | *Lb. sakei hdc* operon | 793 | 100% | 0 | 99% | DQ132888.1 |
|  |  | *Lb. sakei* strain *hdc* gene, partial cds | 793 | 100% | 0 | 99% | AY800122.1 |
|  |  | *T. halophilus hdc* gene *c*omplete and partial cds | 787 | 100% | 0 | 99% | AB670117.1 |
|  |  | *T. muriaticus* plasmid pHDC-I-1 DNA, complete sequence | 787 | 100% | 0 | 99% | AB710473.1 |
|  |  | *O. oeni Hdc operon* | 787 | 100% | 0 | 99% | DQ132887.1 |
|  |  | *Lb. hilgardii hdc* operon | 787 | 100% | 0 | 99% | NG_036021.1 |
| Ossau-Iraty | 8 | *Lb. sakei hdc* operon | 793 | 100% | 0 | 99% | DQ132888.1 |
|  |  | *Lb. sakei* strain *hdc* gene, partial cds | 793 | 100% | 0 | 99% | AY800122.1 |
|  |  | *T. halophilus hdc* gene *c*omplete and partial cds | 787 | 100% | 0 | 99% | AB670117.1 |
|  |  | *T. muriaticus* plasmid pHDC-I-1 DNA, complete sequence | 787 | 100% | 0 | 99% | AB710473.1 |
|  |  | *O. oeni Hdc operon* | 787 | 100% | 0 | 99% | DQ132887.1 |
|  |  | *Lb. hilgardii hdc* operon | 787 | 100% | 0 | 99% | NG_036021.1 |

Supplementary Table S3: Description of the BLAST analysis conducted on *hdc* clones subjected to Sanger sequencing. Max score, query cover, % identity and the relevant accession numbers are included.

Table S3: Supplementary Table 2: Complete BLAST analysis of *tdc* clones subjected to Sanger sequencing

| **Cheese** | **Clone** | **Top BLAST Hits** | **Max score** | **Query cover** | **E value** | **Identity (%)** | **Accession** |
| --- | --- | --- | --- | --- | --- | --- | --- |
| Irish Artisanal Cheese A | 1 | *E. faecalis* complete genome | 1356 | 100% | 0 | 99% | CP008816.1 |
|  |  | *E. faecalis tdc* operon, complete sequence; and putative amino acid transporter gene, complete cds | 1356 | 100% | 0 | 99% | AF354231.1 |
|  |  | *E faecalis tdc* complete cds | 1345 | 100% | 0 | 99% | KF195933.1 |
| Irish Artisanal Cheese A | 2 | *E. faecalis*, complete genome | 512 | 99% | 1.00E-141 | 99% | CP008816.1 |
|  |  | *E. faecalis tdc* operon, complete sequence; and putative amino acid transporter gene, complete cds | 512 | 99% | 1.00E-141 | 99% | AF354231.1 |
| Irish Artisanal Cheese A | 3 | *Lb. curvatus tdc* gene, complete cds | 1286 | 100% | 0 | 99% | AB086652.1 |
|  |  | *S. thermophilus tdcA* gene | 1280 | 100% | 0 | 99% | FR682467.1 |
| Irish Artisanal Cheese A | 4 | *E. faecalis,* complete genome | 1400 | 100% | 0 | 99% | AE016830.1 |
|  |  | *E. faecalis* , complete genome | 1395 | 100% | 0 | 99% | CP008816.1 |
|  |  | *E. faecalis* strain *tdc* gene, complete cds | 1395 | 100% | 0 | 99% | KF195933.1 |
| Irish Artisanal Cheese A | 5 | *E. faecalis* ATCC 29212, complete genome | 1306 | 100% | 0 | 99% | CP008816.1 |
|  |  | *E. faecalis*, complete genome | 1306 | 100% | 0 | 99% | CP002621.1 |
|  |  | *E. faecalis tdc* operon, complete sequence; and putative amino acid transporter gene, complete cds | 1306 | 100% | 0 | 99% | AF354231.1 |
| Irish Artisanal Cheese A | 6 | *Lb. curvatus tdc* gene, complete cds | 1467 | 100% | 0 | 100% | AB086652.1 |
|  |  | *S. thermophilus tdcA* gene | 1461 | 100% | 0 | 99% | FR682467.1 |
| Irish Artisanal Cheese A | 7 | *E. faecalis* complete genome | 1168 | 100% | 0 | 99% | CP008816.1 |
|  |  | *E. faecalis tdc* operon, complete sequence; and putative amino acid transporter gene, complete cds | 1168 | 100% | 0 | 99% | AF354231.1 |
|  |  | *E faecalis tdc* complete cds | 1157 | 100% | 0 | 99% | KF195933.1 |
| Irish Artisanal Cheese A | 8 | *E. faecalis* complete genome | 1411 | 100% | 0 | 99% | CP008816.1 |
|  |  | *E. faecalis tdc* operon, complete sequence; and putative amino acid transporter gene, complete cds | 1411 | 100% | 0 | 99% | AF354231.1 |
|  |  | *E faecalis tdc* complete cds | 1400 | 100% | 0 | 99% | KF195933.1 |
| Reblochon | 1 | *Lb. curvatus tdc* gene, complete cds | 1471 | 100% | 0 | 100% | AB086652.1 |
|  |  | *S. thermophilus tdcA* gene | 1465 | 100% | 0 | 99% | FR682467.1 |
| Reblochon | 2 | *E. faecalis* *tdc* gene, complete cds | 1519 | 100% | 0 | 100% | KF195933.1 |
|  |  | *E. faecalis*, complete genome | 1519 | 100% | 0 | 100% | HF558530.1 |
|  |  | *E. faecalis*, complete genome | 1513 | 100% | 0 | 99% | AE016830.1 |
| Reblochon | 3 | *Lb. curvatus tdc* gene, complete cds | 1330 | 100% | 0 | 99% | AB086652.1 |
|  |  | *S. thermophilus tdcA* gene | 1325 | 100% | 0 | 99% | FR682467.1 |
| Reblochon | 4 | *S. thermophilus tdcA* gene | 1772 | 99% | 0 | 99% | FR682467.1 |
|  |  | *Lb. curvatus tdc* gene, complete cds, | 1772 | 99% | 0 | 99% | AB086652.1 |
| Reblochon | 5 | *Lb. curvatus tdc* gene, complete cds | 1528 | 99% | 0 | 100% | AB086652.1 |
|  |  | *S. thermophilus tdcA* gene | 1522 | 99% | 0 | 99% | FR682467.1 |
| Reblochon | 6 | *Lb. curvatus tdc* gene, complete cds | 1585 | 100% | 0 | 99% | AB086652.1 |
|  |  | *S. thermophilus tdcA* gene | 1580 | 100% | 0 | 99% | FR682467.1 |
| Irish Artisanal Cheese B | 1 | *Lb. curvatus tdc* gene, complete cds | 1495 | 100% | 0 | 99% | AB086652.1 |
|  |  | *S. thermophilus tdcA* gene | 1489 | 100% | 0 | 99% | FR682467.1 |
| Irish Artisanal Cheese B | 2 | *Lb. curvatus tdc* gene, complete cds | 1351 | 100% | 0 | 99% | AB086652.1 |
|  |  | *S. thermophilus tdcA* gene | 1345 | 100% | 0 | 99% | FR682467.1 |
| Irish Artisanal Cheese B | 3 | *S. thermophilus tdcA* gene | 1402 | 100% | 0 | 99% | FR682467.1 |
|  |  | *Lb. curvatus tdc* gene, complete cds, | 1402 | 100% | 0 | 99% | AB086652.1 |
| Irish Artisanal Cheese B | 4 | *Lb. curvatus tdc* gene, complete cds | 1600 | 100% | 0 | 99% | AB086652.1 |
|  |  | *S. thermophilus tdcA* gene | 1594 | 100% | 0 | 99% | FR682467.1 |
|  |  | *Lb. curvatus* partial *tdc* gene | 972 | 65% | 0 | 97% | FN392115.1 |
| Irish Artisanal Cheese B | 5 | *Lb. curvatus tdc* gene, complete cds | 1493 | 100% | 0 | 100% | AB086652.1 |
|  |  | *S. thermophilus tdcA* gene | 1487 | 100% | 0 | 99% | FR682467.1 |
| Irish Artisanal Cheese B | 6 | *Lb. curvatus tdc* gene, complete cds | 1546 | 100% | 0 | 99% | AB086652.1 |
|  |  | *S. thermophilus tdcA* gene | 1541 | 100% | 0 | 99% | FR682467.1 |
| Irish Artisanal Cheese B | 7 | *Lb. curvatus tdc* gene, complete cds | 1472 | 100% | 0 | 99% | AB086652.1 |
|  |  | *S. thermophilus tdcA* gene | 1467 | 100% | 0 | 99% | FR682467.1 |
| Irish Artisanal Cheese B | 8 | *Lb. curvatus tdc* gene, complete cds | 1469 | 100% | 0 | 100% | AB086652.1 |
|  |  | *S. thermophilus tdcA* gene | 1463 | 100% | 0 | 99% | FR682467.1 |
| Tête de Moine | 1 | *E. faecalis* *tdc* gene, complete cds | 1476 | 100% | 0 | 100% | KF195933.1 |
|  |  | *E. faecalis*, complete genome | 1476 | 100% | 0 | 100% | HF558530.1 |
|  |  | *E. faecalis*, complete genome | 1471 | 100% | 0 | 99% | AE016830.1 |
| Tête de Moine | 2 | *Lb. brevis* genome | 1373 | 100% | 0 | 99% | CP005977.1 |
|  |  | *Lb. brevis*, complete genome | 1373 | 100% | 0 | 99% | AP012167.1 |
|  |  | *Lb.plantarum* *tyr*DC and *tyr*P genes, complete cds | 1373 | 100% | 0 | 99% | JQ040309.1 |
| Tête de Moine | 3 | *E. faecalis* *tdc* gene, complete cds | 1567 | 100% | 0 | 100% | KF195933.1 |
|  |  | *E. faecalis*, complete genome | 1567 | 100% | 0 | 100% | HF558530.1 |
|  |  | *E. faecalis*, complete genome | 1561 | 100% | 0 | 99% | AE016830.1 |
| Tête de Moine | 4 | *E. faecalis* *tdc* gene, complete cds | 970 | 100% | 0 | 99% | KF195933.1 |
|  |  | *E. faecalis*, complete genome | 970 | 100% | 0 | 99% | HF558530.1 |
|  |  | *E. faecalis*, complete genome | 965 | 100% | 0 | 99% | AE016830.1 |
| Tête de Moine | 5 | *E. faecalis* *tdc* gene, complete cds | 1587 | 100% | 0 | 99% | KF195933.1 |
|  |  | *E. faecalis*, complete genome | 1587 | 100% | 0 | 99% | HF558530.1 |
|  |  | *E. faecalis*, complete genome | 1581 | 100% | 0 | 99% | AE016830.1 |
| Tête de Moine | 6 | *E. faecalis* *tdc* gene, complete cds | 1417 | 100% | 0 | 99% | KF195933.1 |
|  |  | *E. faecalis*, complete genome | 1417 | 100% | 0 | 99% | HF558530.1 |
|  |  | *E. faecalis*, complete genome | 1411 | 100% | 0 | 99% | AE016830.1 |
| Tête de Moine | 7 | *E. faecalis* *tdc* gene, complete cds | 1448 | 100% | 0 | 99% | KF195933.1 |
|  |  | *E. faecalis*, complete genome | 1448 | 100% | 0 | 99% | HF558530.1 |
|  |  | *E. faecalis*, complete genome | 1443 | 100% | 0 | 99% | AE016830.1 |
| Tête de Moine | 8 | *E. faecalis* *tdc* gene, complete cds | 1421 | 100% | 0 | 99% | KF195933.1 |
|  |  | *E. faecalis*, complete genome | 1421 | 100% | 0 | 99% | HF558530.1 |
|  |  | *E. faecalis*, complete genome | 1415 | 100% | 0 | 99% | AE016830.1 |
| Pecorino Sardo | 1 | *E. faecium*, complete genome | 1275 | 100% | 0 | 99% | CP006620.1 |
|  |  | *E. faecium*, complete genome | 1275 | 100% | 0 | 99% | CP004063.1 |
|  |  | *E. faecium* *tyrS* gene, partial cds; *tyrDC* genes, complete cds, *tyrP* gene, partial cds | 1269 | 100% | 0 | 99% | HM921050.1 |
| Pecorino Sardo | 2 | *E. faecium*, complete genome | 1544 | 100% | 0 | 99% | CP006620.1 |
|  |  | *E. faecium*, complete genome | 1544 | 100% | 0 | 99% | CP006620.2 |
|  |  | *E. faecium* *tyrS* gene, partial cds; *tyrDC* genes, complete cds, *tyrP* gene, partial cds | 1533 | 100% | 0 | 99% | HM921050.1 |
| Pecorino Sardo | 3 | *E. faecium tyrS* gene, partial cds, *tyrDC* genes, complete cds, *tyrP* gene, partial cds | 276 | 85% | 2.00E-70 | 79% | HM921050.1 |
|  |  | *E. faecium*, complete genome | 270 | 85% | 1.00E-68 | 79% | CP006620.1 |
|  |  | *E. faecium*, complete genome | 270 | 85% | 1.00E-68 | 79% | CP004063.1 |
| Pecorino Sardo | 4 | *E. faecium tyrS* gene, partial cds, *tyrDC* genes, complete cds, *tyrP* gene, partial cds | 859 | 97% | 0 | 89% | HM921050.1 |
|  |  | *E. faecium*, complete genome | 843 | 97% | 0 | 89% | CP006620.1 |
|  |  | *E. faecium*, complete genome | 843 | 97% | 0 | 89% | CP004063.1 |
| Pecorino Sardo | 5 | E. faecium, complete genome | 1419 | 100% | 0 | 99% | CP006620.1 |
|  |  | *E. faecium*, complete genome | 1419 | 100% | 0 | 99% | CP004063.1 |
|  |  | *E. faecium* *tyrS* gene, partial cds; *tyrDC* genes, complete cds, *tyrP* gene, partial cds | 1408 | 100% | 0 | 99% | HM921050.1 |
| Pecorino Sardo | 6 | *E. faecium tyrS* gene, partial cds, *tyrDC* genes, complete cds, *tyrP* gene, partial cds | 1142 | 100% | 0 | 99% | HM921050.1 |
|  |  | *E. faecium*, complete genome | 1131 | 100% | 0 | 99% | CP006620.1 |
|  |  | *E. faecium*, complete genome | 1131 | 100% | 0 | 99% | CP004063.1 |
| Pecorino Sardo | 7 | E. faecium, complete genome | 1613 | 100% | 0 | 99% | CP006620.1 |
|  |  | *E. faecium*, complete genome | 1613 | 100% | 0 | 99% | CP004063.1 |
|  |  | *E. faecium* *tyrS* gene, partial cds; *tyrDC* genes, complete cds, *tyrP* gene, partial cds | 1607 | 100% | 0 | 99% | HM921050.1 |
| Ossau-Iraty | 1 | *E. faecalis*, complete genome | 1467 | 100% | 0 | 99% | CP008816.1 |
|  |  | *E. faecalis*, complete genome | 1467 | 100% | 0 | 99% | CP004081.1 |
|  |  | *E faecalis tdc*, complete sequence; and putative amino acid transporter gene, complete cds | 1467 | 100% | 0 | 99% | AF354231.1 |
| Ossau-Iraty | 2 | *E. faecalis*, complete genome | 1823 | 99% | 0 | 99% | CP003726.1 |
|  |  | *E. faecalis*, complete genome | 1823 | 99% | 0 | 99% | CP002621.1 |
|  |  | *E faecalis tdc*, complete sequence; and putative amino acid transporter gene, complete cds | 1807 | 99% | 0 | 99% | AF354231.1 |
| Ossau-Iraty | 3 | *E. faecalis*, complete genome | 1201 | 100% | 0 | 100% | AE016830.1 |
|  |  | *E. faecalis*, complete genome | 1195 | 100% | 0 | 99% | CP008816.1 |
|  |  | *E faecalis tdc*, complete sequence; and putative amino acid transporter gene, complete cds | 1195 | 100% | 0 | 99% | KF195933.1 |
| Ossau-Iraty | 4 | *E. faecalis*, complete genome | 1596 | 100% | 0 | 99% | CP003726.1 |
|  |  | *E. faecalis*, complete genome | 1596 | 100% | 0 | 99% | CP002621.1 |
|  |  | *E faecalis tdc*, complete sequence; and putative amino acid transporter gene, complete cds | 1585 | 100% | 0 | 99% | AF354231.1 |
| Ossau-Iraty | 5 | *E. faecalis tdc* gene, complete cds | 1557 | 100% | 0 | 99% | KF195933.1 |
|  |  | *E. faecalis*, complete genome | 1557 | 100% | 0 | 99% | HF558530.1 |
|  |  | *E. faecalis*, complete genome | 1552 | 100% | 0 | 99% | AE016830.1 |
| Ossau-Iraty | 6 | *Lb. curvatus tdc* gene, complete cds | 1227 | 100% | 0 | 99% | AB086652.1 |
|  |  | *S. thermophilus tdcA* gene | 1221 | 100% | 0 | 99% | FR682467.1 |
|  |  | *Lb. curvatus* partial *tdc* gene | 715 | 61% | 0 | 98% | FN392115.1 |
| Ossau-Iraty | 7 | *Lb. curvatus tdc* gene, complete cds | 1448 | 100% | 0 | 99% | AB086652.1 |
|  |  | *S. thermophilus tdcA* gene | 1443 | 100% | 0 | 99% | FR682467.1 |

Supplementary Table S3: Description of the BLAST analysis conducted on *tdc* clones subjected to Sanger sequencing. Max score, query cover, % identity and the relevant accession numbers are included.

Supplementary Table 4: Total reads assigned for each cheese

| ***hdc* Reads Assigned** | **Reblochon** | **Irish Artisanal Cheese B** | **Morbier** | **Tete de Moine** | **Pecorino Sardo** | **Ossau-Iraty** | **Total Reads** |
| --- | --- | --- | --- | --- | --- | --- | --- |
| **Phylum** | 179002 | 139353 | 231711 | 26719 | 173218 | 188968 | **938971** |
| **Order** | 75790 | 59451 | 73165 | 5371 | 64645 | 25895 | **304317** |
| **Genus** | 3445 | 4764 | 2770 | 527 | 4500 | 0 | **16006** |
| **Species** | 3444 | 3147 | 2770 | 522 | 4496 | 0 | **14379** |
|  |  |  |  |  |  |  |  |
| ***tdc* Reads Assigned** | **Irish Artisanal Cheese A** | **Reblochon** | **Irish artisanal cheese B** | **Tete de Moine** | **Pecorino Sardo** | **Ossau-Iraty** | **Total Reads** |
| **Phylum** | 112469 | 109410 | 81689 | 131959 | 83961 | 105478 | **624966** |
| **Order** | 42581 | 62869 | 44828 | 43726 | 79858 | 50993 | **324855** |
| **Genus** | 19286 | 1355 | 1682 | 32550 | 67084 | 32662 | **154619** |
| **Species** | 9224 | 972 | 1403 | 15495 | 8297 | 890 | **36281** |

Table S4: List of reads assigned at Phylum, Order, Genus and Species level for individual cheeses, post quality filtering.

Supplementary Table 5a: Microbial composition for individual cheeses

| ***hdc* Microbial Composition** | **Reblochon** | **Irish artisanal cheese B** | **Morbier** | **Tete de Moine** | **Pecorino Sardo** | **Ossau-Iraty** |
| --- | --- | --- | --- | --- | --- | --- |
| **Phylum** |  |  |  |  |  |  |
| *Firmicutes* | 100% | 100% | 100% | 100% | 100% | 100% |
| **Order** |  |  |  |  |  |  |
| *Lactobacillales* | 42.34% | 41.38% | 31.58% | 20.10% | 37.32% | 13.70% |
| *Bacillales* | 0% | 1.28% | 0% | 0% | 0% | 0% |
| Unassigned | 57.66% | 57.34% | 68.42% | 79.90% | 62.68% | 86.30% |
| Sum | 100% | 100% | 100% | 100% | 100% | 100% |

Supplementary Table 5b

| ***tdc* Microbial Composition** | **Irish artisanal cheese A** | **Reblochon** | **Irish artisanal cheese B** | **Tete de Moine** | **Pecorino Sardo** | **Ossau-Iraty** |
| --- | --- | --- | --- | --- | --- | --- |
| **Phylum** |  |  |  |  |  |  |
| *Firmicutes* | 100% | 100% | 100% | 100% | 100% | 99.84% |
| *Actinobacteria* | 0% | 0% | 0% | 0% | 0% | 0.16% |
| Sum | 100% | 100% | 100% | 100% | 100% | 100% |
| **Order** |  |  |  |  |  |  |
| *Lactobacillales* | 37.86% | 57.46% | 54.88% | 33.14% | 95.11% | 48.1% |
| *Actinomycetales* | 0% | 0% | 0% | 0% | 0% | 0.16% |
| Unassigned | 62.14% | 42.54% | 45.12% | 66.86% | 4.89% | 51.74% |
| Sum | 100% | 100% | 100% | 100% | 100% | 100% |

Table S5a/b: Microbial composition of bacteria at phylum, order, genus and species levels. Table 5a reflects *hdc* samples while table 5b depicts *tdc* samples.

Supplementary Figure 6: Microbial composition at Genus and Species levels

|  |  |  |  |  |  |  |
| --- | --- | --- | --- | --- | --- | --- |
| ***hdc* samples** | **Reblochon** | **Irish artisanal cheese B** | **Morbier** | **Tete de Moine** | **Pecorino Sardo** | **Ossau-Iraty** |
| **Genus** |  |  |  |  |  |  |
| *Lactobacillus* | 100% | 62.55% | 100% | 100% | 93.07% | 0% |
| *Streptococcus* | 0% | 0% | 0% | 0% | 6.93% | 0% |
| *Staphylococcus* | 0% | 37.45% | 0% | 0% | 0% | 0% |
| **Species** |  |  |  |  |  |  |
| *Lactobacillus buchneri* | 100% | 94.03% | 100% | 100% | 93.06% | 0% |
| *Staphylococcus saprophyticus* | 0% | 5.97% | 0% | 0% | 0% | 0% |
| *Streptococcus thermophilus* | 0% | 0% | 0% | 0% | 6.94% | 0% |
|  |  |  |  |  |  |  |
| ***tdc* samples** | **Irish artisanal cheese A** | **Reblochon** | **Irish artisanal cheese B** | **Tete de Moine** | **Pecorino Sardo** | **Ossau-Iraty** |
| **Genus** |  |  |  |  |  |  |
| *Enterococcus* | 90.80% | 50.63% | 7.67% | 89.31% | 75.32% | 99.65% |
| *Lactobacillus* | 9.20% | 49.37% | 92.33% | 10.69% | 24.68% | 0.35% |
| **Species** |  |  |  |  |  |  |
| *Enterococcus faecalis* | 94.53% | 34.57% | 0% | 95.28% | 2.29% | 100% |
| *Enterococcus faecium* | 0% | 0% | 0% | 0% | 29.37% | 0% |
| *Lactobacillus brevis* | 2.12% | 0% | 0% | 1.94% | 68.34% | 0% |
| *Lactobacillus curvatus* | 0.30% | 65.43% | 100% | 0% | 0% | 0% |
| *Lactobacillus delbrueckii* | 0% | 0% | 0% | 2.79% | 0% | 0% |

Table S6: Relative abundance of bacteria at Genus and Species levels for individual cheeses. Relative abundance is expressed as a function of total reads assigned at the genus level.
